# The Problematic Experience of Players' Mutations Between Clubs: Discovering the Social Adaptability Skills Required

**DOI:** 10.3389/fspor.2021.591438

**Published:** 2021-12-09

**Authors:** Samuel Owiti, Denis Hauw

**Affiliations:** Department of Psychology, University of Lausanne, Lausanne, Switzerland

**Keywords:** adaptability, club transition, experience, situated approach, psychosocial attributes

## Abstract

**Objective:** During their career, most players working in professional team sports move from club to club. These transitions are not always completely successful and could highly impact the route of the players' development. However, there is a lack of knowledge on the psychological processes involved when players encounter problems in adapting from one club to another. Thus, it was the aim of this study to identify the most difficult aspects of these transitions, as experienced by team sports players and the psychological skills that contribute to successful outcomes.

**Design and Method:** The present study included twenty professional basketball players (aged between 20 and 36 years old; Mean = 26.05, SD = 4.12), who had played under different coaches (coach range 4–15; Mean = 8.65, SD = 2.92), and also played for different clubs (range 3–10; Mean = 5.35, SD = 2.08). They took part in retrospective interviews regarding their embedded experiences during club to club transitions. A situated E-approach was used to identify their problematic experiences, the adaptability skills and how they are applied during club mutations.

**Results and Conclusions:** The identification of problematic experiences revealed seven components in relation to coaching (e.g., obeying orders, reduced play time), three components with teammates (e.g., respect), two components with the club (e.g., lack of support), and three components with family/friends (e.g., geographical constraints). Additionally, results indicated that the adaptability skills used during mutation are related to three groups namely mental skills, learning methods, and interpersonal skills. The results provide coaches, players, sports psychologists, and national sport organizations a set of issues for understanding the challenges players encounter when they move from one club to another.

## Introduction

Looking at the career of players in team sports, one key point is the success of their mutation between clubs. Indeed, it is unusual for a player to spend their whole career at the same club and these transitions are important events in most professional team sports (e.g., NBA basketball drafts, Mercato transfers in soccer, and Major league volleyball drafts). Besides the economic issues and effects of these moves, questions of how a players' sporting life adapts to new teammates, new coaches, new training forms and new cultures and geographical settings remain (Owiti et al., 2020). It is surprising that the impact of the new relationships caused by mutation between clubs has tended to be overlooked in sport psychology to date (Vaeyens et al., 2008; Owiti et al., 2020). There is a lack of knowledge relating to the experience of mutation, especially in terms of the level of success and the psychological adaptations required. The key dimensions of the environment that affect these outcomes are as yet unelucidated. In parallel, the processes of adaptation experienced by players when they move to a new club remain unclear. For example, the duration of their adaptation and the psycho-social dynamics at stake have not been explored in-depth to date. It was thus the aim of the current study to examine the psychological processes linked to these adaptations during players' club mutation. We hypothesize that this adaptation is a psycho-social process requiring specific skills (labeled Social Adaptability Skills) because adapting effectively and achieving a successful mutation is a competency embedded in the specificities of the professional sports environment.

## Mutation as a Contextual Transition

A mutation changes the relationships of the players with their sporting environment. It combines a variety of contextual components that could be characterized within the microsystems, mesosystems, exosystems, and macrosystems in which a person is constantly embedded (Bronfenbrenner, 1979; Krebs, 2009). Sport psychology studies have investigated the relationships between players and various components of their microsystem in-depth. This kind of knowledge could be informative for understanding what is at stake during club mutations, such as their effects on peers or teammates (e.g., Allen, 2003; Höll and Burnett, 2014; Erickson and Côté, 2016; Garn, 2016), coaches (e.g., Jowett and Poczwardowski, 2007; Rhind et al., 2012; Jowett, 2017), or parents and family (e.g., Horn and Horn, 2007; Clarke and Harwood, 2014; Knight et al., 2016; Pynn et al., 2019). Research to date has characterized how the components of a players environment influence their development by supporting (e.g., players stability and harmony), constraining (e.g., undermining players well-being), accelerating (e.g., increasing the performance success and satisfaction), or modifying how a player handles the impact of a problematic situation. In addition, their results could inform us about the potential issues for mutation and how to solve them, however, in a mutation, all of these components of the microsystem act together as a whole and a combined consideration of them is required to better understand this specific question.

Club mutations have been considered in studies that have investigated the relationships with the environment involved at the level of a macrosystem (i.e., transitions over the career of an athlete) (e.g., Wylleman and Lavallee, 2004; Wylleman et al., 2004; Stambulova et al., 2009, 2012; Drew et al., 2019). In this framework, a series of normative transitions during an athletes' career (Wylleman and Lavallee, 2004; Stambulova et al., 2009), at the end of the career (Alfermann and Stambulova, 2007; Park et al., 2012; Blijlevens et al., 2018), and non-normative transitions such as injury have been identified (Ivarsson et al., 2018). If the processes of adaptation linked to these transitions could also be informative when considering mutations, it is less a normative transition than a micro-phase included in the general phases of transition that is at stake (mainly in the mastery phase for professional players and sometimes in the development phase for talented players considering the Holistic Athletic Career model (Wylleman and Lavallee, 2004). It is not a non-normative transition because the mutation is predictable and could be anticipated. Therefore, the career transitions framework provides elements of context for the transition. It shapes the importance of the issues of the outcomes by contextualizing the mutation in the timeline of a career.

Mutations between clubs seem particularly complex to study because of how players relate to a changing environment that includes people (microsystem), culture (macrosystem), interactions (mesosystems), and links to social setting (exosystem) (Bronfenbrenner, 1979; Krebs, 2009). Various other components impact changes in the environment when players move between clubs: club structure (i.e., management, philosophy, equipment), coaches, teammates, family, friends (distance), and culture (other people, habits, languages, geography, climate). Therefore, at this stage, we define mutation as changes from club to club that occur in the professional player's career during the mastery phase (Wylleman and Lavallee, 2004), as they are seldom involved in the development phase of talented players.

Mutations can have various levels of outcomes, set in a continuum from total success to complete failure. These could be studied by focusing on the analysis of players' experiences which represent one of the key components of the evaluation of mutation outcomes besides the assessment of members of staff for example. Indeed, the player is often best placed to evaluate themselves when deciding on the success of a mutation. The others can have an opinion but they do not always know all the dimensions that are at stake in this assessment. For example, it is difficult to know exactly how the players live in terms of their perception of being distant from home because they do not necessarily reveal this type of intimate experience to staff in a professional work environment, and they may anticipate being at risk of being penalized or stigmatized. In addition, analysis of the difficult sides of the mutation may reveal what is required for successful outcomes because it highlights the different types of perturbation that players have to face in this situation that remain hidden when club mutations go well. This approach focuses on the analysis of players' problematic situations or critical incidents in activity that are relevant for revealing the processes and adaptations that are successful in various types of sporting situations (Hauw and Durand, 2007; Hanton et al., 2008; Villemain and Hauw, 2014; Kostamo et al., 2019).

## Adaptations in Mutation

We hypothesize that when moving to a new club, adaptations consist of a series of psycho-social processes that impact the interaction between the player and the new environment. Situated and Enactive approaches to human activities (often referred to by the label “Four E Approach of human activity,” including Embedded, Embodied, Extended, and Enacted) suggest that adaptation could be described as reciprocal adaptations (Bronfenbrenner, 1979; Varela, 1996; Bruner, 2000; Krebs, 2009; Rowlands, 2010). The adaptations transform the new environment into a familiar and personal situation that could be grasped via the meaningful world of athletes and players (Hauw, 2018; Owiti et al., 2020). It means that in mutation, the environment influences the players and the players transform their environment meaningfully to re-establish a sense of equilibrium. These adaptations have been studied in a variety of sports situations underlying the plasticity of the worlds of meanings that are linked to the athletes' activity (e.g., D'Arripe-Longueville et al., 2001; Gouju et al., 2007; Mottet and Saury, 2013; Gesbert et al., 2017; Rochat et al., 2017; Hauw, 2018). Adaptation reflects the conception of a player as an active agent in, and on, their environment. As a result of the uncertainty and the dynamic nature of the relationship between players and their environment, the outcomes of these adaptations might lead to inconsistencies, various unresolved situations and finally, the player dropping out of the sport system (Schlossberg, 1984; Pulakos et al., 2000; Côté et al., 2009; Hauw, 2017; Hauw and Bilard, 2017; Stambulova, 2017). It is also well-known that each component of the players' environment may provide different opportunities for actions (i.e., affordances, artifacts, or resources) because each player can be transformed by meaningful and personal experiences (e.g., those that experience successful or unsuccessful transformation). If this transformation is successful, then the personal situation is linked to positive experiences, positive well-being, and sport performance progression (Edwards et al., 2004; Côté et al., 2009; Light, 2010; Davis and Jowett, 2014; Passos et al., 2016). In contrast, problematic experiences in the new environment could lead to isolation and crisis thereby bringing a set of demands usually appraised as stressors to the player (Alfermann and Stambulova, 2007; Stambulova and Wylleman, 2014). Additionally, considering the multiple components of the environment, it might not be surprising to observe different processes of adaptation at the same time such as good interaction with the coach but not with teammates. This is an example of what (Bronfenbrenner, 1979; Krebs, 2009) describe as a “battle of dispositions” between developmentally-generative and developmentally disruptive attributes within the same environment.

Further to the four E approaches, mutations could also be considered within some social psychology frameworks that have addressed the dynamic aspects of life changes (e.g., Zittoun, 2009). Here the focus posits that changes in the development of a person involve constantly changing adjustments between their environment, going through a series of relatively stable periods, alternating with brutal ruptures (Van Geert, 2003; Zittoun, 2009). During the moments in which the person's development is interrupted, reoriented, or challenged, the person develops new conduct to answer the challenges through a process of transition. A mutation is typically this kind of transition: in the better case, it reorientates the career of the players such as gaining a new position in a team; in the worst-case scenario it interrupts their development, for example when a player experiences reduced play time. Thus, in this framework, transitions have been defined as processes of catalyzed change due to rupture, aiming at a new sustainable fit between the person and their current environment (Zittoun et al., 2003). The notion of rupture has been defined in different manners, *irritation* (Peirce, 1878), *crisis* (Vygotsky, 2004), *desequilibration* (Piaget, 2003), or *challenge* (Smelser and Erikson, 1980). Therefore, mutations can be seen as processes of reorganization of a system in which the person's interpretation of a perceived rupture plays a major role in their subsequent thoughts and actions. Additionally, the mutation could be understood within a range of ruptures that should be identified.

Thus, in these various frameworks, the processes of adaptation comprise actions done by players to reorganize the equilibrium between themselves and their situations. All these actions are situated because they are linked to the experience of the moment, of the environment, and what is felt by the players. When these actions are successful, one can consider that the player has developed a competency that could be used in an equivalent situation. This is what we call a skill (Danish et al., 1996; Danish and Nellen, 1997) and because the mutation required is a psycho-social process of adaptation, we called these skills Social Adaptability Skills. As a working definition, social adaptability skill is the capacity learnt during mutations to adapt efficiently to a problematic experience.

Considering the importance of successful mutations and the complexity of related adaptations, the current study's aims were two-fold: (a) to identify problematic meaningful experiences encountered by players during mutation from club to club; (b) to identify social adaptability skills and attributes that are valuable during players mutation between clubs. To achieve this we designed the study with an enactive approach that identifies meaningful experiences from inter-team moves by interviewing players who have changed between clubs.

## Methods

### Methodological Congruence

Holt and Tamminen (2010) suggested that qualitative studies should display “*methodological congruence*,” that is, consistency should be evident throughout the research question, epistemological perspective, and ontological viewpoint. Our study is based on a four E approach (i.e., Embedded, Embodied, Extended, and Enacted) to human activity that frames analysis of the dynamic interactions between a person and their environment, and underlines an enacted consideration of the environment (i.e., the situation). This epistemological perspective is based on a self-organized ontology of human beings centered on conception of the human as creating their own situations, in their singularities, by selecting what is important for them within different possibilities (Maturana and Varela, 1980; Mingers, 1991; Joas, 1996; Kupiec and Sonigo, 2000). This also suggests a consideration of the analysis of experience as one of the pillars of the enactive understanding of how a person builds their own situations (e.g., Hauw, 2018). This could be done by simultaneously analyzing what is done in a context and what is meaningful for the person. Previous research in sport studies has used this methodological congruence and we align the design of this study within this framework.

#### Participants

In the current study, participants who had the most appropriate experience with respect to the research question were initially recruited through purposive sampling. Elite basketball players were recruited since this population encounters a large number of club to club mutations during their career progression (CIES Observatory, 2019). The names and addresses of all the players were obtained through contacts from the Swiss Basketball Federation, which is the governing sports body currently covering 17,000 members from its nine regional associations comprising 185 clubs. A total of 35 basketball players were emailed a cover letter explaining the purpose of the study out of which 25 players responded positively. Five players could not be interviewed due to time and distance constraints. Finally, the study involved a convenient sample of twenty European and American professional basketball players (age range 20–36; Mean 26.05, SD = 4.12). There was a total of (*n* = 17) male, and (*n* = 3) female participants. Overall, twelve players had gone through European basketball academies in their respective countries (for example in France, Swiss, and Lithuania) and had performed at the highest national levels (Swiss Basketball League, League National Basketball France). On the contrary, eight remaining participants had gone through the North American academies (for example in the USA and Canada) and had played in one of the highest leagues (the National Basket Association G League and Women's National Basketball Association). For players to be included in the study, they must be currently playing at an international/professional level and have also gone through more than one club mutation (club range 3–10; Mean 5.35, SD = 2.08). Finally, the players must have trained under more than one coach (coaches' range 4–15; Mean = 8.65, SD = 2.92).

#### Data Collection

Ethical approval was granted from the Ethics Committee of the University of Lausanne (Project number: E_SSP_062021_00001) and informed consent was obtained from all participants. Interviews were conducted on an individual basis using a semi-structured interview guide. We sought to help the players evoke their personal experiences during different club mutations. We thus organized the interview setting in agreement with the participants, allowing them to feel relaxed, comfortable and available for the evocation process. Initially, we proposed a first part of the interview to build a timeline of the mutations, this required us to decompose the flow of time into periods to keep the semiotic trace of the adaptability experience. Conceptually, the interview guide focused on a player's timeline, incorporating all the mutations from club to club to facilitate the understanding of the dynamics of personal experience in-depth and in relation to specific elements of the environment of each club. This methodology has been used in doping studies and has shown relevance in eliciting personal experiences (e.g., Hauw and Lemeur, 2013; Hauw and Bilard, 2017). More specifically, the current study focused on experience starting with entry into a club environment and continuing to the present day. The timeline (x-axis) was used to support the interviews and guide the participant to be open about their experiences and has also been shown to increase the accuracy and insights in retrospective recall (Drasch and Matthes, 2013).

Secondly, the following part of the interview focused on the experience for each period of the timeline. The interview guide format consisted of five main question areas to reflect the recommendations of Morgan and Krueger (1998). These areas included: (i) opening questions- involved creating a thoughtful, permissive atmosphere and setting the tone for the interview; (ii) *introductory questions*, which had the purpose of gaining the participant's attention, introducing the topic and explaining its relevance to the study. For example, the question: “in order to describe your experience of the mutation in this club, how would you describe your relationship with the coach?;” (iii) *key questions* that focused on answering the aims of the current research. For example: “did you find the experience problematic?,” “What did you find meaningful in the experience?,” “How did you overcome the problematic experience?;” (iv) *transition questions* moving from one period to the next. For example: “what has changed now as compared to the previous experience?;” and (v) *ending questions*, which involved asking the participant to reflect on the entire discussion and invited them to offer their positions or opinions on the central topic. For example: “is there any additional comment you would like to reflect on?”

The interview guide consisted of the following series of areas and dimensions of experience reflected in the six key introductory questions: (i) how would you describe your relationship with the coach? (ii) How would you describe your relationship with the teammates? (iii) How would you describe the feeling of being away from your family? (iv) What was your general feeling about the club? (v) How would you describe adapting to the coaching style? (vi) How did you adapt to the coaching expectations? Finally, we asked the players to rate their experience of the transition at the end of the club mutation, and whether they thought that the mutation was either successful or problematic. From an applied and practical standpoint, the interviews we conducted were based upon a pilot interview conducted with one elite basketball player. The narrative length, which ranged from 4,798 to 12,861 words and lasted between 30 and 90 min, were digitally recorded in their entirety.

#### Data Analysis

**Figure 1:**
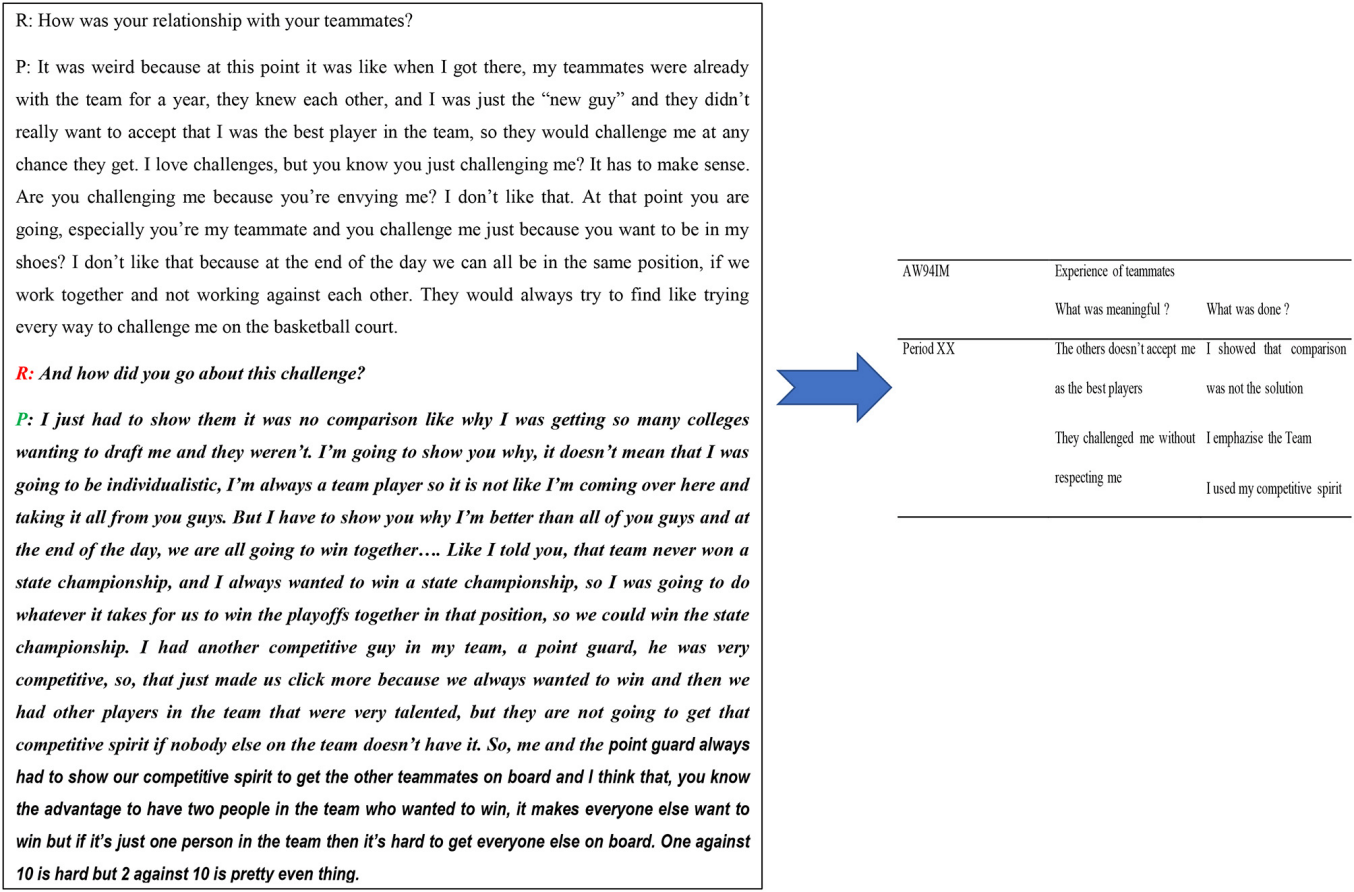
Enactive coding example.

Of the 20 participant interviews, we only chose episodes that displayed the problematic situations faced by a player during a club mutation. We first identified problematic situations during mutation then listened again to the interviews. A problematic situation was identified by looking at disturbances in the transition that players reported as being difficult enough to prevent them from performing at their best (e.g., responses such as “I just could […] not adapt to [the] coaching style.” “I was always lonely and feeling homesick from being away from my family,” or “I found the level of play was low and the practices lacked structure”). Further selection was performed to identify the problematic aspects of the mutation discussed by the participants, after which they were transcribed verbatim. Of the 20 participants included, we only extracted problematic experiences and these using four categories, including teammates, coach, family, and club. The data analyzed therefore comprised the following aggregated problematic experiences: 27 cases with teammates, 38 cases with a coach, 15 cases with family, and 27 cases with a club. Additionally, we aggregated the number of times each participant experienced either a successful or problematic mutation using the four categories (teammates, coach, family, and club). To analyse the adaptation process, the described experience was split into two parts (what was meaningful and the events/examples that took place in this situation) (see Figure 1 for an example). This approach was adapted from a previous coding system used for the analysis of experiences in various sports situations (Hauw et al., 2003; Hauw and Durand, 2007, 2008; Hauw and Bilard, 2017; Rochat et al., 2017, 2018; Hauw, 2018).

To conduct a comparison of the participant responses, all the individual codings were compressed into more general categories using inductive content analysis (Biddle et al., 2001). Each experience identified (i.e., what was meaningful and what was done) was grouped into more general dimensions. As the coding progressed, the coding themes emerging from the data from one of the transcripts informed the coding of the following. Constant comparative method was applied (Weed, 2009; Holt and Tamminen, 2010). It involved developing additional codes, identifying emerging themes within the data and constantly comparing the codes in myriad ways (for example between participants or between different periods). Through a process of feedback and discussion, the coders came to a unanimous decision regarding each coded theme. In accordance with the criterion of theoretical saturation (Strauss and Corbin, 1998; Weed, 2009; Holt and Tamminen, 2010; Corbin and Strauss, 2015), data collection and analysis were discontinued when the categories upon which the theory was built no longer produced new insights. At the end of data processing, we obtained a list of problematic meaningful experiences and the actions/events relating to them. These results were considered as types of experience and the “adaptability skills” deployed by players.

## Results

This section first presents the types of problematic meaningful experiences encountered by players during mutation from club to club and secondly reports on actions undertaken in these situations, which relate to the “adaptability skills” deployed by players regarding their experiences.

**Figure 2:**
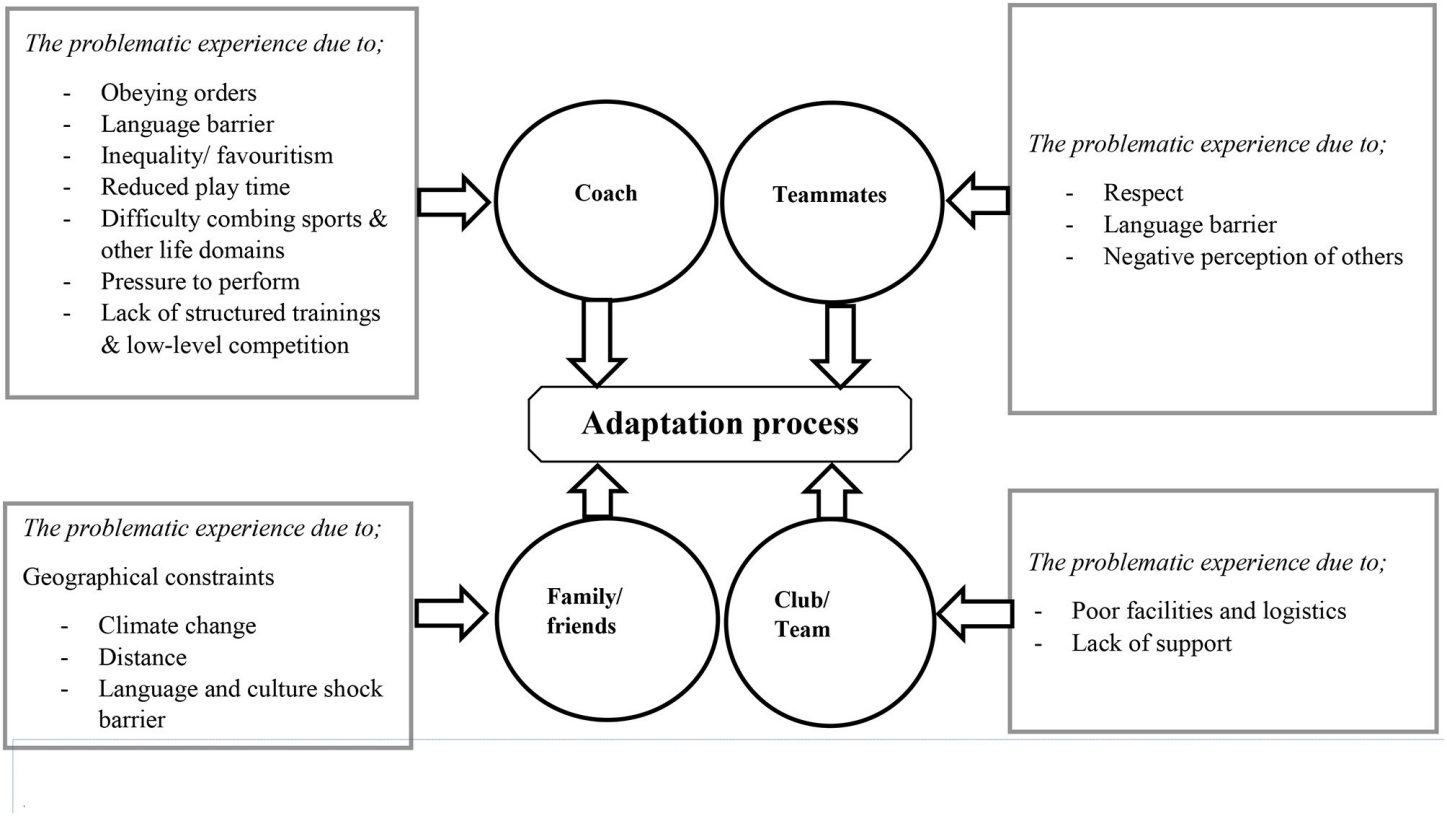
An overview of the problematic meaningful experience of athletes during club mutations in relation to the four categories. The four circles represent the categories used to explore the problematic experiences during mutation targeted in this study. The four rectangles represent the sub-themes indicating the different types of problematic meaningful experiences.

Figure 2 represents the different types of problematic meaningful experiences experienced by players during club mutation. We observed seven problematic experiences involving the coach, three with teammates, two with the club/team, and three concerning family and friends (see Figure 2).

### Problematic Meaningful Experiences With Coaches

This category contained both problematic experiences arising from coach expectation and coaching style. Thus, problematic experiences of the coach-player relationship were described by the following sub-categories: obeying orders, language barrier, inequality/favoritism, reduced play time, difficulty combining sports and other life domains, pressure to perform, lack of structured training, and low level competition.

#### Obeying Orders

Players admitted that to adapt within the team setting, they developed specific approaches to obeying orders or following instructions. As a result of these demands, some players displayed problematic experiences in conforming to or obeying orders and found arguments to justify their feelings, as indicated by this example:

“I think it was just my personality. I'm an intense person, so whenever the coach instructed us to do things, I would be hard headed and wouldn't want to do it straight and that would be it. This led to the coach losing confidence in me since I wouldn't listen to his instructions and from then on, my play time reduced considerably” (participant CD94IF).

#### Language Barrier

Players admitted that effective communication within the team brought an established rapport between coaches and players, which in turn had a significant role in contributing to players adaptability. However, there were instances of a communication breakdown, as reported by participant AR88IM:

“It was not easy for me and the other players because the coach was an Italian who didn't speak any French language at all. He would often come to me or the other guy who also spoke Italian language to translate his directives to the other team members. We found this to be really bizarre since it literally made communication with him difficult” (participant AR88IM).

#### Inequality/Favoritism

Some participants highlighted cases of inequality, which they say hindered their adaptation process. Participant AW94IM observed that the “coach had his son plus those whom he treated as favorite players on the team, of course you are going to want the best for your child, but you can't jeopardize, you can't discourage other players from their dreams.”

#### Reduced Play Time

The players reported that reduced play time led to negative affects including emotional reactions such as crying and feeling frustrated. This in turn made the players experience low self-esteem and anxiety, as outlined by participant AW94IM:

“I wasn't on the starting list. I wasn't the type that was going to voice like how frustrated I was. It's always frustrating when you are always playing and then like, now, I'm not playing anymore, you ask yourself what is going on? You can play it down but down deep me, I knew I was frustrated and mad” (participant AW94IM).

#### Difficulty Combining Sports and Other Life Domains

There were cases where the players found themselves negotiating between sports development and studies.

“It was difficult, my schedule was exhausting. There were times I would go for training from 12 to 14 h then go have lunch, I would then go for my class before heading back for my basketball practice for like 4 h practice and that was like for two seasons. I really never got a break and at some point, it was too much, and I had to quit” (participant CD94IF).

#### Pressure to Perform

Participants reported that some coaches focused solely on results, resulting in players experiencing pressure to perform. An American player expressed this, outlining how:

“I was always frustrated since we were the only two professionals in the team,while the others were either students or semi-professionals. The coach would always expect us the professionals to do everything in the court be it scoring, playing defence, attacking. On top of that, you had to score a lot of points if you really wanted the club to renew your contract the following year” (participant EU94IM).

#### Lack of Structured Training and Low-Level of Competition

In some instances, overseas players reported finding the level of practice and competitions not challenging enough, as stated by participant MM93IF:

“It was a big shock. I acknowledge never to have expected that (low-level), coming in you think that I'm playing professionally and every year I'm going to step up my career. I would rate my college basketball higher than my current professional team in Europe. It's difficult to adapt because the challenge here is just not enough” (participant MM93IF).

### Problematic Experience With Teammates

The analyses of problematic meaningful experiences were grouped into three sub-categories namely: lack of respect, language barrier, and negative perception of others.

#### Respect

There were reported cases of players searching for respect and approval from teammates, as discussed by participant:

“It was weird because at this point my teammates were already with the team for a year, they knew each other, and I was the ‘new guy’ and they didn't really want to accept that I was the best player in the team, so they would challenge me. I had to show them why I was the highest paid draft” (participant AH95IM).

#### Language Barrier

There were cases of players not being able to communicate with fellow teammates as narrated by participant CD94IF:

“I moved to Romania to play basketball, we were the only two players from the USA who were speaking English, culturally speaking, it was different. There was no communication at all, apart from gestures, we could speak to them in English and they would respond in their language which we didn't understand at all. The misunderstanding brought a lot of conflicts on court and I would say we were all to blame for not having made an effort to learn one another's language. The bonding and even the effort to do so was zero. Can you imagine going for a drink with your teammates if you can't communicate? It's just not possible” (participant CD94IF).

#### Negative Perception of Others

There were cases where participants felt unwelcome in their new club by fellow teammates and this slowed or complicated the adaptability process. Additionally, there were players who reported that the type of relationships they had with their teammates was “conditional” (i.e., based on image, money, and/or performance). This was discussed by participant BH97IM, who outlined that the “relationship I felt with my teammates was that of sport friends. It was a very formal and rigid type of relationship. No time for human side talks, it doesn't have to be about basketball all the time. I missed that human side of it.”

### Problematic Experience Due to Family/Friends

This category provided a general theme linked to geographical and cultural constraints. Participants underlined the distance, climate change, language and culture shock barrier. A couple of the players had to quit their homes to join the basketball academies at a young age or to play overseas.

#### Language and Culture Shock Barrier

There were a couple of players who encountered problematic feelings of language and culture shock barrier.

“I was always missing home, I mean it's lonely here because I don't have things to do like I would do back home, and people are not used to getting together here. I'm somebody who loves being surrounded by people wherever I go. You don't realize how much you miss home, like in the bus when I hear people speak French language and you just feel like it's you and a bunch of other people around you, there is no connection” (participant MM93IF).

#### Climate Change

As concerns climate change, participant AH95IM reported that:

“Having lived in Texas for many years, I enjoyed the hot weather which was favorable, however, when I came to Switzerland, and to make it worse, in early January […] right in the middle of winter, freezing temperatures. My hands were always cold and painful to a point that I didn't enjoy going out there for practices. I mean, this snow thing is not really meant for me.” (participant AH95IM).

#### Distance

The time difference factor impacted participant CD94IF negatively, who reported that:

“I'm from Chicago, so I'm used to those freezing temperatures, my main issue was that, I couldn't really communicate with my friends and family back home. The time difference couldn't just allow it. My mum who works in a bank would be at work already in the morning while I'm at my practices or games. I need regular communication with my sister, mother, and my friends. Without my family, and not being able to communicate with them, the world feels empty. All these just made me feel homesick and bored in my apartment” (participant CD94IF).

### Problematic Experience Due to Club/Team

This category involved two sub-themes, including poor facilities and logistics, and lack of support. These represent structures that facilitate the adaptability of players. The focus was more on the environment in which the players' adaptation took place. The mutation environment had the sports team as the core, but it also went beyond the players' direct interaction with the club (e.g., facilities and financial support) and related to supporting players' adaptability.

#### Lack of Support

A lack of support was mostly connected to low budgets, meaning several were clubs not able to provide the necessary support needed by the players. This was reported by participant JW93IM:

“I was used to traveling on nice buses, taking flights and leaving the night before for away games so you have good rest before the games. At my current club, it is a complete adjustment for me. Now, I have to wake up by 7 h then travel about 4 h to go play then come back, that's not easy because my recoveries are very compromised” (participant JW93IM).

#### Poor Facilities and Logistics

Concerns about poor facilities and logistics were detrimental to players adaptability. Participant SL87IM observed that

“The club did not have enough basketball court for practices, there were times when we could start practices only after 22 h. We would then finish very late and by the time you reach home exhausted, it's way past midnight. The worst part was during winter, the practice hall was so cold because the heating system had broken down and it took months and months before it finally got repaired” (participant SL87IM).

### Adaptability Skills

This section reports on the types of adaptability actions (i.e., what they did) and the corresponding skills deployed by players during club to club changes. To link this to situations, we contextualized the adaptability actions with the problematic experiences of players during mutations (see Table 1).

**Table 1:** Types of adaptability actions and skills deployed by players during club mutations.

**Problematic experience**	**Adaptability actions**	**Adaptability skills**
**Coach**		
- Obeying orders	Willing to be disciplined	Self-discipline
- Language barrier	Willing to interact with coach and support staff	Interpersonal skills
- Inequality/favoritism	Willing to accept challenges (sense of involvement)	Motivation/confidence, positive thinking
- Reduced play time	Accepting challenges to progress	Goal setting, positive thinking
- Difficulty combining sports & other life domain	Wanting to balance between sports and other life domain	Self-organization
- Pressure to perform	Using challenges to progress	Motivation/confidence, positive thinking
- Lack of structured training & low-level competition	Having knowledge of motives to participate in sports (intrinsic, competitiveness)	Motivation/confidence, goal-setting
**Teammates**		
- Respect	Respecting others	Interpersonal skills,
- Language barrier	Willing to interact with teammates	
- Negative perception of others	Willing to interact with fellow teammates	
**Club/Team**		
- Poor facilities and logistics	Reacting to hard times positively	Positive thinking, motivation/confidence
- Lack of support	Willing to step out of comfort zone	
**Family/friends**		
- Climate change	Taking responsibility for own development	Self-discipline, motivation/confidence, autonomy
- Distance		
- Language and culture shock barrier		

To overcome problematic situations, players described the actions they used, which refer to seven adaptability skills: self-discipline, goal setting, motivation/confidence, self-organization, interpersonal skills, positive thinking, and autonomy.

#### Self-Discipline

The actions reported by the players at this level focused strictly on rules, following planification, ignoring the negative experience, and staying focused on the set goals. Participant CD94IF reported:

“I think it (self-discipline) probably came from me, I think I finally became like very coachable, like I would just listen to what the coach had to say then try and do it. Like I wouldn't fight the situation. I think because I was getting paid as a professional now and I'm getting older too” (participant CD94IF).

#### Goal Setting

The actions reported by the players were to build a strategy to find a solution to a difficult experience. It comprised analyzing the situation, identifying goals and sub-goals and making a strategy to attain them, including self-assessment. Players reported pursuing a range of both short and long-term goals during adaptations, as was in this case for participant EU95IM:

“When there is a new coaching style that I need to adapt to, I set some goals of how to achieve it, I keep reviewing the progress and evaluate the whole process, this I do quite often with my coach to see where I am, with this, I don't mind the repetition, it just means that I have achieved my goal once the repetition comes to an end. And on repetition, you just have to get used to it if it means adapting to my coach. There are hard things out there way tougher than this, so you just have to play along” (participant EU95IM).

#### Motivation and Confidence

The actions reported by the players at this level referred to being aware of the motives for participating in sports, for example, intrinsic motivation and competitiveness. In this study context, motivation and confidence were closely linked, as described by participant VG93IM:

“You have to believe in yourself and go for it. If you have the desire, do not hold back even if it involves challenges like I had to go overseas, just don't stop. In sports, changing of environment is part of the game, so this should not hold you back. So, if your dreams are to become professional, then you have to be ready to adapt to different challenges” (participant VG93IM).

#### Self-Organization

The actions that were reported by the players included staying focused on different tasks (i.e., balancing between sports, studies, and/or work) and maintaining social life effectively. These methods were re-iterated by participant VG93IM:

“First, joining that club was a big change for me. The idea was that I wanted to study sports science at the university because playing basketball in Swiss (Switzerland) is good but it's not something which can support your life in terms of a salary. I was searching for a club near me, and a club which was ready to accept that I don't train every day so that I can get time off for my studies. I negotiated with the club that I do not practice during lunch hour and they were ok with it” (participant VG93IM).

#### Interpersonal Skills

At this level, the actions reported by the players involved communication and interaction with people at the club (i.e., coaches, teammates, management staff). Participant BH97IM discussed this issue of team bonding:

“I'm probably the biggest advocate for it (bonding with teammates), I think obviously in any sport but especially team sport, high pressure and where all the players have to be on the same page like in basketball, I think knowing each other within the sport and personally is one of the most important things especially throughout the season where it is very long and there are no roller coasters, there are ups and downs, if you are friends off the court, nothing will be able to break you on the court” (participant BH97IM).

#### Positive Thinking

The actions reported by the players at this level referred to a willingness to accept challenges through a sense of involvement, reacting to hard times positively, and using challenges to progress, as reported by participant EU94IM:

“I know the budget is small here as compared to other rich clubs, yes, so sometimes we have to do those back and forth trips to the games. Hey, listen, I'm here to play basketball, I'm here to perform, so, I really don't pay much attention to how those things will affect me. What's important for me is that the club gives me a chance to make myself better, perform better, other things are secondary. It's all about focus” (participant EU94IM).

#### Autonomy

In terms of autonomy, the players reported actions such as building a strategy in taking responsibility for their development. This comprised the players' belief that one's actions are self-directed, hence players feeling in control of their own behaviors and goals as reported by participant VG93IM:

“It was not challenging. I didn't get any problems with that (being away from family) because it had already been 1 year since I was living away from them. The time I was at (club) helped me be independent, so, I didn't miss my parents. I think when I first left home at 14 years, there were two things, I wanted to go but again a bit hard when you think of leaving the family cocoon, then once at the academy, it's not like we forget about the family but it's all about playing basket and committing 100%” (participant VG93IM).

## General Discussion

The purpose of this investigation was two-fold: (a) to identify problematic meaningful experiences encountered by players during mutation from club to club; and (b) to identify the social adaptability skills and attributes that are valuable during players mutation between clubs.

Overall, in contrast with the French movie *Life Is a Long Quiet River* (*La vie est un long fleuve tranquille*) by Chatiliez (1988), our results showed that mutations from club to club are not a “*longue fleuve tranquille*” and require psychosocial resources for a successful outcome and co-adaptation. This spectrum of challenges are linked to the new club's system of functioning, including coaches' attitudes, habits and types of coaching, and ways of negotiating possible relationships with teammates, including accessibility and openness to culture as well as clubs resources for training and performing. This spectrum also includes the family dependency or autonomy of the player, which enables them to cope with being away from home. Club mutations subject players to a set of perturbations that affect the microsystem, mesosystems, and key psychosocial balance or equilibrium of a player in a club (Bronfenbrenner, 1979). These perturbations affect the players' experience because they create a discrepancy with the habits, knowledge or expectations of the player (Vygotsky, 2004). Each player comes to a new club with a set of standards or norms regarding what is possible and what is tolerable in a club. For example, in our results, the attitudes of a coach were surprising for participant AW94IM because they was not prepared to encounter inequality and favoritism as manifested by the coach. In addition, participant EU94IM got frustrated by the fact that the coach expected them to do everything in the court putting immense pressure on performance. These two examples show that each respective player had little experience with different coaches. Therefore, the mutation challenges engaged at the higher level include frustration tolerance regarding the norms of what is acceptable and openness or a capacity to aggregate new knowledge and adapt. This reinforces the field of promoted activity that these players have built when they interact with a new environment (Reed, 1993; Valsiner, 1997, 2001).

Frustration, tolerance, and openness to new experiences are first linked to a players' personality (i.e., facets of the Big Five). They may play a determinant role in the ease with which they adapt to mutation constraints because when people score high in these facets, they show easiness when facing these types of challenges, even if they have never encountered them before. However, according to the multi-levels of identity (McAdams, 1996; McAdams and Olson, 2010), this is not the only possibility. Indeed, if a person does not score high at this level of personality, skills, as well as narratives, may play a role in compensation (e.g., McAdams and Pals, 2006; Soto, 2021) and thus be involved in successful adaptations (Owiti et al., 2020, 2021; Hauw and Carrière, 2021).

Our study identified the specific actions and skills players have learnt that enable them to achieve successful mutations. The skills identified in our study could be split into three groups. The first group gathered general mental strategies that are used to face constraining environments such as goal setting, motivational or focusing attitude (e.g., The Ottawa Mental Skill Assessment Tool OMSAT-3 (Durand-Bush et al., 2001). A second group focused on skills that target the learning methods per see such as self-discipline or self-organization (Vealey, 1988; Orlick, 1992). At this level, the players re-centered their dispositions to act wishing it to help them meet the expectation of the coach or reduce the constraints of the club for achieving in the specific context of a successful mutation. A third group of skills is related to interpersonal skills. This was not specified in the current listing of mental skills for elite athletes (e.g., OMSAT-3). However, it seems to play an important role in adaptation in mutation. These are also identified by Soto (2021) as key skills that are important for talent development (MacNamara and Collins, 2011) and efficient learning and coaching (Jowett, 2017).

In the discussion that follows, we focus on the examination of the four general categories expected to cause problematic experiences during club to club mutations.

### Problematic Experiences With Coach and Adaptations

An athlete's performance is constrained by the circumstances of competing for the availability of resources, which once obtained offer possibilities for success (Passos et al., 2016). This defines the athlete-coach relationship, which has been found to be influential during mutation as well as in both social and athletic development (Jowett, 2005; Jowett and Poczwardowski, 2007; Smith and Smoll, 2007). Our analysis revealed that players perception of satisfaction of basic psychological needs generally mediated their relations with the coach. The attachment theory (Ainsworth, 1978), which aims to promote an understanding of the dyads that are formed in close relationships, could offer insight into why some players encounter problematic experiences with the coach. The theory posits that a person's attachment behavior is categorized into three styles: (a) *secure*–evident in persons who display confidence with others in providing them with support and comfort in times of need; (b) *anxious-ambivalent*–displayed in persons who have a strong desire for proximity and intimacy with close other and become frustrated upon separation; and (c) *avoidant attachment*–displayed persons who exhibit little distress and few attempts at maintaining contact with close others. Therefore, players who had a secure attachment style with the coach expected that assistance would be available in times of need. Conversely, players with insecure attachment styles expect inconsistent assistance or no assistance at all (Leak and Cooney, 2001; Carr, 2009; Davis and Jowett, 2010; Carr and Fitzpatrick, 2011; Diehl et al., 2019).

Fundamentally, players perception of the mutation between clubs, and how accurate (or otherwise) this is, may determine the influence that their perceptions have on the outcome. If players perceive the change to be more problematic than it is or underestimate the challenge they are experiencing, they may experience a more problematic process. This line of thought converges with those outlined in previous studies (Jowett and Poczwardowski, 2007; Jones et al., 2014; Olsson and Pehrson, 2014; Morris et al., 2015, 2017; Stambulova et al., 2017; Franck and Stambulova, 2019; Pummell and Lavallee, 2019).

### Problematic Experiences With Teammates and Adaptations

Research on sports performance (Eime et al., 2013) has already established the critical nature of the relationship between teammates. Various models such as Interdependence Theory (e.g., Casper et al., 2007) have provided frameworks for understanding the way teammates relational dyads influence each other based on mutual rewards. The results of the current study confirm the athlete-athlete dyad whereby each individual tries to maximize rewards (happiness, social status, emotional support, and pleasure), while minimizing the costs (anxiety, negative emotions, and conflicts) (Thibaut and Kelley, 2007). It has been suggested that decreasing interpersonal interactions between teammates can disturb intrateam cohesion (Warren, 2006; Jowett, 2017). In our study, we cite an example that was reported by participant BH97IM who felt that the relationship they had with teammates was a formal and rigid “sport friends” type of relationship. In cases where the athlete's relation with environment is challenged or in crisis, utilization of affordances could help the individual to co-adapt by providing opportunities and solutions to achieve successful mutation (Passos et al., 2009, 2013). It is therefore significant that good relationships between teammates are established to help individuals manage stress, cultivate skill development, adapt during mutations, and improve social relationships (Morris, 2013; Nunomura and Oliviera, 2013; Allen and Laborde, 2014; Höll and Burnett, 2014; Passos and Davids, 2015; Passos et al., 2016).

### Problematic Experiences With the Club/Team and Adaptation

In an organization that considers talent development a key component of its culture and values, players experience support during mutation (Williams and Reilly, 2000; MacNamara and Collins, 2011; Morris et al., 2015). Additionally, the environment where a player plays or trains can be highly influential to their development (e.g., Henriksen et al., 2010; Martindale and Mortimer, 2011; Ivarsson et al., 2018). The dimension of how the players felt about the club, in general, encompasses the notion of whether it led to the players' development being facilitated or challenged. We provide an example of participant JW93IM who was concerned about their recoveries being compromised as a result of poor traveling logistics. In addition, participant SL87IM complained about broken facilities at the club, which also led to problematic experiences. Therefore, the level of uncertainty that the players experienced impacted how ready they felt during a change from one club to another (Jones et al., 2014). Our study found that those players who are generally motivated and possess anticipatory positive thinking attitudes were able to adapt during club mutation despite the challenges. This finding supports the Talent Identification Model, which stresses the importance of player development within appropriate environments (Durand-Bush and Salmela, 2002; MacNamara et al., 2010; Côté et al., 2014).

### Problematic Experience of Adapting to Being Away From Family/Friends

As a result of recent technological advances in both communication and travel, crossing land borders for both career and personal life has been facilitated (Schinke et al., 2019). However, players in the current study were faced with multiple challenges (e.g., climate change, distance, language, and culture shock barriers). An example of players who experienced problematic mutations includes participant CD94IF who complained about the time difference impacting negatively their social life, participant AH95IM, who could not tolerate the freezing temperatures in Europe, and finally participant MM93IF who admitted that language and the culture shock barrier compromised their integration. Therefore, for reciprocal interaction to occur, Bronfenbrenner (1999) has suggested that the immediate environment must be of a kind that invites attention, exploration, and imagination. As such, human development takes place within a cultural system that constitutes the context and the reality of the person and how they interact with the communities and social institutions that are both proximal and distal (Tonyan et al., 2013). Players in the current study interact in different contexts and internalize certain cultural values and practices, either facilitating or presenting challenges for club mutation. As Weisner argues, there are multiple behavioral and mental processes involved in the attainment of culture (Weisner, 2005). From this perspective, an individual's culture mentality includes shared and idiosyncratic beliefs, practices, and experiences that can either produce cultural conflict, leading to problematic experiences or positive culture integration and hence, positive experiences. Our findings support the considerable evidence in wider literature that families play a crucial role in socializing individuals into sport (Baker and Horton, 2004; Schinke et al., 2019).

To conclude, our enactive approach has led to the identification of several problematic experiences that forced players to cope with adaptation by developing specific actions. Our results underline the strength of this approach by allowing us to capture what was experienced by players in the specific situations that were meaningful for them at the same time, and how they managed their own experiences by developing specific actions. The current results indicate the overlaps between situation and action (i.e., situated activity; Reed, 1993; Kirshner and Whitson, 1997; Engeström et al., 1999; Sannino and Sutter, 2011) that emerge at the meaningful level of players' experiences during a mutation. They also showed that this approach could also be useful since it has been stated in ergonomics (e.g., Theureau, 2003), social science (e.g., Durand, 2013), and sports psychology research through analyses of performance (e.g., Rochat et al., 2019), competition (e.g., D'Arripe-Longueville et al., 2001; Hauw and Durand, 2007), training (e.g., Hauw, 2018), and doping (Hauw and Mohamed, 2015).

### Limitations and Future Research

Several limitations should be acknowledged, highlighting questions that remain unresolved, and defining potential directions for future research. A systematic analysis using the principles of the enactive analysis approach enabled an understanding of the players' mutations between clubs. However, qualitative studies of this kind are restricted in many ways. Since the purpose of the current study was not to verify hypotheses but to generate new insights from the social adaptability skills demonstrated, these findings are not easily generalizable. The results are context-specific and need to be tested in different contexts, with larger samples to increase the scope. Therefore, it remains of interest as to whether studies with bigger samples show similar outcomes. The semi-structured interviews the participants provided involved retrospective recall and as such are subject to memory bias. Given that there was no representative sampling for the participants, players who experienced problematic mutations might not have been inclined to narrate the truth, or they might have downplayed these difficulties. Therefore, future research could recruit a larger experientially, more diverse, and representative sample population. The study focused exclusively on basketball players at an elite level of sport and does not inform us about the nature of adaptability in other sports or levels. Therefore, future research should widen the scope into various other sports and levels. The predictive validity of the current findings could be investigated longitudinally to allow the capturing of the social adaptability process in real-time and also to identify issues perceived as challenging during particular times. Future studies could also be deployed to ascertain how these adaptation skills can be assessed and developed. Finally, future directions for research should attempt to determine if a dynamic relationship exists between adaptability skills and individual traits by retrospective evaluation.

### Practical Implications

The current results present valuable insights into the social adaptability skills used by players to adapt to club mutations. Individuals working with elite players should identify and monitor these adaptability skills (e.g., self-discipline, goal-setting, motivation/confidence, self-organization, interpersonal skills, positive thinking, and autonomy), which a player needs to develop, and should intervene to help attain the balance between these factors. In addition, players' immediate environment must be carefully managed to foster the development of adaptability skills that will help them overcome challenges. Furthermore, educational programs that focus on how to evaluate and develop these adaptability skills should be entrenched within talent identification and development programs either through formal and/or informal psycho-social training.

The current findings could also contribute to and influence different social adaptability skills in players. For example, a player's ability to set goals and assess personal performances could stimulate their investment during mutations. From a coach's perspective, the current study provides emphasis on development, providing insights that will enable them to contribute positively during player mutations. High performance organizations and professional sports clubs could use the current findings to identify major adaptability challenges and barriers faced by incoming players. Additionally, the sports clubs could use these findings to maintain existing and develop new resources to effectively assist players and support them in the challenges they encounter during mutations.

## Conclusion

Research in the area of career transitions in sport has increased gradually over time, as reflected in the number of studies and reviews. Investigations to date have contributed a better understanding of players' career transition process at a macro-level, but further research is needed at a micro-level. This present study identified the key social adaptability challenges and the social adaptability skills players apply when they move from club to club.

## Data Availability Statement

The raw data supporting the conclusions of this article will be made available by the authors, without undue reservation.

## Ethics Statement

The studies involving human participants were reviewed and approved by Ethics Committee Lausanne University. The patients/participants provided their written informed consent to participate in this study.

## Author Contributions

Both the authors contributed equally to the conception of the current article, with DH providing insights and analysis while SO did the wrote up.

## Conflict of Interest

The authors declare that the research was conducted in the absence of any commercial or financial relationships that could be construed as a potential conflict of interest.

## Publisher's Note

All claims expressed in this article are solely those of the authors and do not necessarily represent those of their affiliated organizations, or those of the publisher, the editors and the reviewers. Any product that may be evaluated in this article, or claim that may be made by its manufacturer, is not guaranteed or endorsed by the publisher.
